# 基于全二维气相色谱-飞行时间质谱分析室内灰尘中邻苯二甲酸酯及其新型替代品

**DOI:** 10.3724/SP.J.1123.2023.12002

**Published:** 2025-02-08

**Authors:** Linxiao WANG, Ke GAO, Jianjia LI, Jiahui PENG, Ziyan YANG, Erken YA, Congyi ZHENG, Wei WEI, Liping LU, Shuiyuan CHENG

**Affiliations:** 北京工业大学区域大气复合污染防治北京市重点实验室,北京100214; Key Laboratory of Beijing on Regional Air Pollution Control, Beijing University of Technology, Beijing 100214, China

**Keywords:** 全二维气相色谱-飞行时间质谱联用, 邻苯二甲酸酯, 室内灰尘, 广谱筛查, comprehensive two-dimensional gas chromatography-time of flight mass spectrometry (GC×GC-TOF-MS), phthalates (PAEs), indoor dust, broad spectrum screening

## Abstract

建立了室内灰尘中25种传统邻苯二甲酸酯(PAEs)及19种新型替代品的全二维气相色谱-飞行时间质谱联用(GC×GC-TOF-MS)分析方法。灰尘样品经正己烷-二氯甲烷(1∶1, v/v)溶液超声萃取30 min后,以Rix-5MS(30 m×0.25 mm×0.25 μm)为一维柱、Rix-17Sil(1.39 m×0.25 mm×0.25 μm)为二维柱进行分离。在最佳实验条件下,可以实现快速、准确、灵敏的室内灰尘中25种传统PAEs和19种新型PAEs替代品的检测。44种目标物在1~500 μg/g范围内呈现良好的线性关系,相关系数均在0.99以上,检出限(LOD)为0.57~13.0 ng/g,在1、10、50 μg/g加标水平下,各待测化合物的回收率为72.8%~125%,相对标准偏差为1.29%~14.8%(*n*=3)。将该方法应用于40份校园室内环境(教室、食堂、实验室、宿舍)灰尘中PAEs及其替代品的分析。结果显示,室内灰尘中PAEs及其替代品的总含量范围为2.07~354 μg/g。其中,邻苯二甲酸二(2-乙基己基)酯(DEHP)是最主要的检出物,含量范围为nd~158 μg/g,其次是PAE新型替代品对苯二甲酸二(2-乙基己)酯(DEHTH),含量范围为nd~117 μg/g。不同室内环境中PAEs及其替代品表现出显著的组分和含量差异,提示室内PAEs具有广泛的来源。该方法简单、快速,精密度、准确性和稳定性良好,并且能够同时检出多种PAEs,适于室内灰尘中PAEs的含量测定,也可为未来室内多功能区PAEs的来源解析及风险评估提供技术参考。

## Abstract (en)

A comprehensive two-dimensional gas chromatography-time-of-flight mass spectrometry (GC×GC-TOF-MS) method was developed to analyze 25 traditional phthalate esters (PAEs) and 19 novel alternatives in indoor dust samples. PAEs are ubiquitous in indoor environments because they are widely used as plasticizers in a variety of consumer products, and potential health concerns have prompted the need for effective monitoring methods. In this study, dust samples were collected from various indoor settings in a university campus, including classrooms, cafeterias, laboratories, and dormitories, and were subsequently ultrasonically extracted with hexane-dichloromethane (1∶1, v/v) solution for 30 min. This method was chosen to maximize PAE recovery while minimizing potential interference from other compounds present in the dust matrix. Compounds were separated on a Rix-5MS column (30 m×0.25 mm×0.25 μm) as the first dimension, with a Rix-17Sil column (1.39 m×0.25 mm×0.25 μm) serving as the second dimension. The following temperature program was used: 60 ℃ for 1 min, then increasing to 220 ℃ at 20 ℃/min, followed by a further increase to 290 ℃ at 5 ℃/min, with the final temperature held for 8 min; this program was optimized to afford maximum target-compound resolution and sensitivity. The developed method rapidly, accurately, and sensitively detected the target PAEs and their alternatives under the optimal conditions, which included a carrier-gas flow rate of 1.4 mL/min, a modulation period of 4 s, and an injection-port temperature of 250 ℃. The 44 target compounds exhibited highly linear calibration curves across a content range of 1-500 μg/g, with all correlation coefficients exceeding 0.99. The limits of detection (LODs) of the method were determined to lie between 0.57 and 13.0 ng/g, which reflects the high sensitivity of the developed approach. At spiked levels of 1, 10, and 50 μg/g, the recoveries of the analyzed compounds varied from 72.8% to 125%, with relative standard deviations ranging from 1.29% to 14.8% (*n*=3), which indicates that the method is highly precise and reliable. The developed method was used to analyze PAEs and their alternatives in 40 indoor dust samples, which revealed total contents of between 2.07 and 354 μg/g in dust samples. Di-2-ethylhexyl phthalate (DEHP) emerged as the most frequently detected compound, with contents ranging from “not detected” (nd) to 158 μg/g. The novel alternative, bis(2-ethylhexy) terephthalate (DEHTH), was also detected, with levels ranging from nd to 117 μg/g. Notably, significant differences in the compositions and contents of the PAEs and their alternatives were observed across various indoor environments, which suggests that diverse sources and exposure pathways exist for these compounds, highlighting the necessity for ongoing PAE monitoring and risk assessment in various indoor settings. In conclusion, the developed GC×GC-TOF-MS method provides a powerful tool for comprehensively analyzing PAEs and their alternatives in indoor dust; it is well-suited for the routine monitoring of these compounds owing to its simplicity, rapidity, and robustness. These findings provide valuable insight for future research into the sources and health implications of PAEs in indoor environments, and ultimately support risk assessment and regulatory efforts.

邻苯二甲酸酯(phthalate esters, PAEs)是一类普遍存在的塑料添加剂,被广泛用于玩具、清洁剂、个人护理用品(如指甲油、头发喷雾剂、香皂和洗发液)等产品中^[1⇓-3]^。由于PAEs与聚合物之间没有化学键合,因此很容易从日常产品中逸出^[4]^,并通过呼吸、皮肤吸收和摄入等暴露途径进入人体^[5]^。目前,PAEs已在人体血清、尿液和母乳中被广泛检出^[1,6,7]^。PAEs暴露会导致多种不良反应,如内分泌干扰、生殖和神经毒性等^[8⇓⇓⇓-12]^。有研究指出,传统PAEs中的邻苯二甲酸二(2-乙基己基)酯(DEHP)暴露会对小鼠的学习、记忆和神经系统造成不利影响^[11]^。由于对传统PAEs毒性作用的日益关注,部分国家或地区开始限制或禁止传统PAEs的使用^[13,14]^,而作为替代品的新型PAEs正在世界范围内大量生产和使用。

二(2-乙基己基)对苯二甲酸二酯(DEHTP)、环己烷-1,2-二羧酸二异壬酯(DiNCH)和二(2-丙基辛基)邻苯二甲酸二酯(DPHP)是DEHP和邻苯二甲酸二异壬酯(DiNP)的3种主要替代品,2017年这些替代品在欧洲市场的生产量高达约100万吨^[15]^。其他PAEs替代品如三聚酯、柠檬酸酯和己二酸酯等,也被广泛应用于社会生活的各个领域^[16]^。目前,这些替代品已经陆续在室内灰尘、空气和颗粒物中被检测到^[17⇓⇓-20]^,其中室内灰尘是大量半挥发性和非挥发性物质的赋存基质,而PAEs类物质为主要检出组分^[21]^,不同国家和地区室内灰尘中均有不同程度的PAEs被检出^[5,15,20]^。例如,乙酰柠檬酸三丁酯(ATBC)在中国、澳大利亚和美国的室内灰尘中检出率为100%,含量范围为0.05~62.5 μg/g,其潜在的暴露风险不容忽视。

虽然已有较多国内外研究报道了PAEs及其替代品在室内灰尘中的赋存特征及其潜在人体健康危害^[15,22⇓-24]^,但是现有检测方法主要针对传统PAEs,新型替代品存在检测方法空缺的问题,针对传统PAEs及其替代品同时检测的方法更加匮乏。尽管有研究者指出新型PAEs替代品对环境污染较少且没有健康危害^[25]^,但是越来越多的研究指出PAEs替代品具有与传统PAEs类似的内分泌干扰活性、生殖和发育毒性、细胞毒性、肝毒性和致癌性等^[26⇓⇓-29]^。现代人90%以上的时间在室内度过^[30]^, PAEs及其替代品在室内环境中的赋存特征及其人体健康风险已成为一个备受关注的问题。更加值得关注的是,某些传统PAEs及其替代品同时存在可能具有效应累积作用^[31]^,在实际样品中同时检测PAEs及其替代品,将有利于全面解析PAEs类物质的人体暴露风险。因此,亟需开发针对室内灰尘中传统PAEs及其替代品同时检测的广谱分析方法。

高效简便的前处理方法是实现广谱分析的重要前提。室内灰尘中有机污染物的分析通常需要利用提取技术进行目标化合物的溶剂提取,常见的方法包括索氏提取、超声辅助提取、加速溶剂提取和微波辅助提取^[32]^。超声萃取是提取灰尘中PAEs及其替代品的常用方法,通过正己烷、二氯甲烷、丙酮等有机试剂的多次超声萃取,可以将灰尘样本中大部分的PAEs及其替代品提取出来^[33⇓-35]^,但是超声萃取需要注意萃取温度和萃取时间^[36]^,从而避免PAEs及其替代品的转化和损失。PAEs及其替代品通常使用气相色谱-质谱法(GC-MS)进行检测^[37⇓-39]^。由于PAEs及其替代品具有相似的理化性质,同时检测几十种PAEs及其替代品将面临分离难题。相比于一维气相色谱,全二维气相色谱(GC×GC)采用调制器连接两个极性不同、分离机理不同的独立色谱柱来实现复杂待测物的正交分离,极大地提高了色谱的峰容量^[40]^。而飞行时间质谱(TOF-MS)具有较高的采集频率,可满足GC×GC对扫描速率的要求,是目前GC×GC常用的高效检测器^[41]^。Hurtado-Fernández等^[42]^利用GC×GC-TOF-MS对汽车灰尘中的半挥发性有机物进行非靶向筛查,成功检测到多环芳烃、氯化/溴化芳香族化合物、有机磷阻燃剂、PAEs、酚类、苯并三唑类等245种化合物,但该研究并没有对PAEs及其替代品进行精确的定性和定量分析。

本研究基于超声萃取和GC×GC-TOF-MS技术,通过优化超声萃取的时间和次数、GC×GC-TOF-MS的进样口温度、离子源和传输线温度、升温程序、PAEs及其替代品的前处理和色谱-质谱参数等进行了优化,建立了同时检测灰尘中25种PAEs及19种替代品的简单、快速、灵敏度高的分析方法,并应用于40个实际室内灰尘样本中44种目标物的研究。

## 1 实验部分

### 1.1 仪器、试剂与材料

Pegasus 4D GC×GC-TOF-MS (美国LECO公司)。25种PAEs及19种替代品的标准品(具体名称见表1,纯度≥98%)购自上海阿拉丁公司,5种同位素内标己二酸二(2-乙基己基)酯-D_8_(DEHA-D_8_)、邻苯二甲酸二正庚酯-D_4_(DnHP-D_4_)、邻苯二甲酸二乙酯-D_4_(DEP-D_4_)、邻苯二甲酸二辛酯-D_4_(DnOP-D_4_)、邻苯二甲酸二甲酯-D_4_(DMP-D_4_)(具体名称见表1,纯度≥98%)均购自加拿大TRC公司,无水硫酸钠购自上海阿拉丁公司,二氯甲烷和正己烷(均为色谱纯)购自美国Thermo Fisher公司;Milli-Q超纯水(18.2 MΩ·cm)。

**表1:** 25种PAEs、19种替代品和5种同位素内标的全称、缩写、分子式、特征离子和保留时间

No.	Compound		Abbreviation		Formula		Characteristic ions (*m/z*)		Internal standard		Retention Ⅰ/s		Retention Ⅱ/s	
	PAEs													
1	di-2-ethylhexyl phthalate (邻苯二甲酸二(2-乙基己基)酯)		DEHP		C_24_H_38_O_4_		149^*^, 104, 57		DEHA-D_8_		1008		1.96	
2	butyl benzyl phthalate (邻苯二甲酸丁苄酯)		BBzP		C_19_H_20_O_4_		149^*^, 93, 65		DEP-D_4_		880		2.59	
3	dibutyl phthalate (邻苯二甲酸二丁酯)		DBP		C_16_H_22_O_4_		149^*^, 76, 41		DEP-D_4_		640		1.36	
4	diisobutyl phthalate (邻苯二甲酸二异丁酯)		DiBP		C_16_H_22_O_4_		149^*^, 121, 65		DEP-D_4_		620		1.29	
5	dicyclohexyl phthalate (邻苯二甲酸二环己酯)		DCHP		C_20_H_26_O_4_		149^*^, 93, 65		DEP-D_4_		1000		2.79	
6	diethyl phthalate (邻苯二甲酸二乙酯)		DEP		C_12_H_14_O_4_		149, 93^*^, 65		DEP-D_4_		520		1.03	
7	dihexyl phthalate (邻苯二甲酸二己酯)		DHxP		C_20_H_30_O_4_		149, 121^*^, 76		DEP-D_4_		868		1.92	
8	diisohexyl phthalate (邻苯二甲酸二异己酯)		DiHxP		C_20_H_30_O_4_		149, 121^*^, 76		DEP-D_4_		844		1.85	
9	dimethyl phthalate (邻苯二甲酸二甲酯)		DMP		C_10_H_10_O_4_		163, 133^*^, 77		DMP-D_4_		472		1.03	
10	diisopropyl phthalate (邻苯二甲酸二异丙酯)		DiPrP		C_14_H_18_O_4_		149^*^, 43, 41		DMP-D_4_		544		1.03	
11	di-*n*-propyl phthalate (邻苯二甲酸二正丙酯)		DPrP		C_14_H_18_O_4_		149^*^, 105, 76		DMP-D_4_		584		1.22	
12	di-*n*-heptyl phthalate (邻苯二甲酸二正庚酯)		DnHP		C_22_H_34_O_4_		149^*^, 104, 57		DnOP-D_4_		948		1.98	
13	diisoheptyl phthalate (邻苯二甲酸二异庚酯)		DiHP		C_22_H_34_O_4_		149^*^, 104, 57		DnOP-D_4_		964		2.01	
14	di-*n*-octyl phthalate (邻苯二甲酸二正辛酯)		DnOP		C_24_H_38_O_4_		149, 93^*^, 65		DnOP-D_4_		1184		1.91	
15	dinonyl phthalate (邻苯二甲酸二壬酯)		DNP		C_26_H_42_O_4_		149, 104, 55^*^		DnOP-D_4_		1144		2.18	
16	diisononyl phthalate (邻苯二甲酸二异壬酯)		DiNP		C_26_H_42_O_4_		293, 149, 71^*^		DEP-D_4_		1216		2.18	
17	diundecyl phthalate (邻苯二甲酸二十二烷基酯)		DUP		C_30_H_50_O_4_		149^*^, 121, 55		DEP-D_4_		1616		3.67	
18	diallyl phthalate (邻苯二甲酸二烯丙酯)		DAlP		C_14_H_14_O_4_		149, 132^*^, 104		DnHP-D_4_		576		1.25	
19	diamyl phthalate (邻苯二甲酸二戊酯)		DAmP		C_18_H_26_O_4_		149, 104^*^, 76		DnHP-D_4_		756		1.7	
20	diisopentyl phthalate (邻苯二甲酸二异戊酯)		DiPeP		C_18_H_26_O_4_		149, 104, 71^*^		DnHP-D_4_		712		1.56	
21	isobutylcyclohexyl phthalate (邻苯二甲酸异丁基环己酯)		iBCHP		C_18_H_24_O_4_		223^*^, 150, 149		DnHP-D_4_		776		1.96	
22	diphenyl phthalate (邻苯二甲酸二苯酯)		DPhP		C_20_H_14_O_4_		225, 77^*^, 65		DnHP-D_4_		1020		3.52	
23	didecyl phthalate (邻苯二甲酸二异癸酯)		DiDP		C_28_H_48_O_4_		307, 149, 65^*^		DEHA-D_8_		1192		1.95	
24	diphenyl isophthalate (间苯二甲酸二苯酯)		DPiP		C_20_H_14_O_4_		225, 104^*^, 76		DnOP-D_4_		1136		3.35	
25	dibenzyl phthalate (邻苯二甲酸二苄酯)		DBzP		C_22_H_18_O_4_		149, 91^*^, 61		DEHA-D_8_		1176		3.91	
	PAE alternatives													
26	acetyl tri-*n*-butyl citrate (乙酰柠檬酸三丁酯)		ATBC		C_20_H_34_O_8_		185, 129^*^, 57		DEHA-D_8_		812		1.65	
27	bis(2-ethylhexyl) adipate (己二酸二(2-乙基己基)酯)		DEHA		C_22_H_42_O_4_		129^*^, 83, 55		DEHA-D_8_		900		1.51	
28	diheptyl, *n*-nonyl adipate (己二酸庚基壬基酯)		DHeNoA		C_22_H_42_O_4_		129^*^, 111, 55		DEHA-D_8_		832		1.48	
29	diisobutyl adipate (己二酸二异丁酯)		DiBA		C_14_H_26_O_4_		129, 111^*^, 57		DEHA-D_8_		548		0.91	
30	diisodecyl adipate (己二酸二异癸烷基酯)		DiDeA		C_26_H_50_O_4_		129^*^, 111, 57		DEHA-D_8_		1096		1.59	
31	trioctyl trimellitate (偏苯三酸三辛酯)		TOTM		C_33_H_54_O_6_		305, 193^*^, 57		DEHA-D_8_		1772		1.61	
32	bis(2-ethylhexy) terephthalate		DEHTH		C_24_H_38_O_4_		167, 149^*^, 70		DEHA-D_8_		1108		1.98	
	(对苯二甲酸二(2-乙基己)酯)													
33	bis(2-ethyl-1-hexyl) isophthalate		BEHIP		C_24_H_38_O_4_		149, 112^*^, 57		DEHA-D_8_		1152		1.86	
	(间苯二甲酸二异(2-乙基己基)酯)													
34	tributyl trimellitate (偏苯三酸三丁酯)		TBTM		C_21_H_30_O_6_		249, 193, 165^*^		DEHA-D_8_		1772		0.12	
35	di(2-ethylhexyl) cyclohexane-1,2-dicarboxylate		1,2-DEHCH		C_24_H_44_O_4_		155^*^, 71, 57		DEHA-D_8_		968		1.71	
	(二(2-乙基己基)环己烷-1,2-二羧酸酯)													
36	di(2-ethylhexyl) tetrahydrophthalate		DEHT		C_24_H_42_O_4_		152^*^, 124, 57		DEHA-D_8_		972		1.75	
	(二(2-乙基己基)四氢邻苯二甲酸酯)													
37	di-isodecyl 4-cyclohexene-1,2-dicarboxylate		DiDCH		C_28_H_50_O_4_		124, 85^*^, 57		DEHA-D_8_		1228		1.91	
	(4-环己烯-1,2-二羧酸二异癸酯)													
38	di-methyl-isophthalate (间苯二甲酸二甲酯)		DMIP		C_10_H_10_O_4_		163^*^, 77, 50		DEHA-D_8_		472		1.1	
39	*n*-butyryl-tri-(*n*-hexyl)-citrate		BTHC		C_28_H_50_O_8_		157^*^, 129, 71		DEHA-D_8_		1288		1.97	
	(正丁基柠檬酸三(正己基)酯)													
40	trimethyl citrate (柠檬酸三甲酯)		TMC		C_9_H_14_O_7_		143^*^, 101, 59		DEHA-D_8_		468		1.03	
41	tributyl citrate (柠檬酸三丁酯)		TBC		C_18_H_32_O_7_		129^*^, 101, 57		DEHA-D_8_		772		1.59	
42	triethyl citrate (柠檬酸三乙酯)		TEC		C_12_H_20_O_7_		157, 115^*^, 87		DEHA-D_8_		540		1.01	
43	dibutyl adipate (己二酸二丁酯)	DBA		C_14_H_26_O_4_		129^*^, 111, 55		DEHA-D_8_		576		1.01	
44	di-isononyl cyclohexane-1,2-dicarboxylate	DINCH		C_26_H_48_O_4_		155, 109^*^, 81		DEHA-D_8_		472		1.13	
	(环己烷-1,2-二羧酸二异壬酯)												
	Internal standards												
45	bis(2-ethylhexyl) adipate-D_8_	DEHA-D_8_		C_22_H_42_O_4_		137^*^, 83, 57				896		1.5	
	(己二酸二(2-乙基己基)酯-D_8_)												
46	di-*n*-heptyl phthalate-D_4_ (邻苯二甲酸二正庚酯-D_4_)	DnHP-D_4_		C_22_H_34_O_4_		153, 108^*^, 80				868		1.91	
47	diethyl phthalate-D_4_ (邻苯二甲酸二乙酯-D_4_)	DEP-D_4_		C_12_H_14_O_4_		153, 125^*^, 80				520		1.03	
48	di-*n*-octyl phthalate-D_4_ (邻苯二甲酸二正辛酯-D_4_)	DnOP-D_4_		C_24_H_38_O_4_		153^*^, 108, 55				1144		2.17	
49	dimethyl phthalate-D_4_ (邻苯二甲酸二甲酯-D_4_)	DMP-D_4_		C_10_H_10_O_4_		167, 137^*^, 81				468		1.07	

Retention Ⅰ: retention time of the 1^st^ dimensional chromatography; Retention Ⅱ: retention time of the 2^nd^ dimensional chromatography; * quantitative ion.

### 1.2 标准溶液配制

精确移取44种PAEs及其替代品混合标准母液,用正己烷-二氯甲烷(1∶1, v/v)溶液稀释,配制含量为1000 μg/g的混合标准溶液储备液。内标混合标准溶液用正己烷-二氯甲烷(1∶1, v/v)溶液进行稀释,配制含量为40 μg/g的内标混合标准溶液。所有溶液均保存在-20 ℃冰箱中。

将44种PAEs及其替代品混合标准溶液用正己烷-二氯甲烷(1∶1, v/v)溶液稀释,加入定量内标混合标准溶液,配制成含量分别为1、5、10、50、100、200、500 μg/g的混合标准溶液,其中内标含量为20 μg/g。

### 1.3 样品采集和前处理

采用主动采样法,在吸尘器金属钢管头部套上尼龙采样袋,用吸尘器吸取北京市某高校校园环境中实验室(*n*=10)、宿舍(*n*=10)、教室(*n*=10)和食堂(*n*=10)地面的灰尘,采样后小心取下采样袋,用绳子封好口并用干净的铝箔纸将采样袋包裹好,放入自封袋内。采样前将采样袋用二氯甲烷和超纯水超声清洗3次,每次采样后用甲醇对金属钢管进行清洗,避免样品污染。随后,将采样袋中的灰尘用150目筛子过筛,并将过筛后的灰尘用铝箔包裹好,保存在-20 ℃冰箱中,待处理。

前处理方法参考文献[34]并进行了改进:称取2 mg灰尘于15 mL玻璃试管中,加入混合内标40 ng,室温下静置3 h。向试管中加入2.5 mL正己烷-二氯甲烷(1∶1, v/v)溶液,涡旋2次,每次1 min。然后,将试管放入水浴中超声萃取30 min,每次超声后取上清液于玻璃试管,以4500 r/min离心6 min,离心后取上清。重复3次提取样品,合并后的上清液在氮气流下浓缩吹至近干,加入200 μL正己烷-二氯甲烷(1∶1, v/v)溶液复溶并振荡。将溶液过0.22 μm有机滤膜,转移至2 mL进样瓶内,待GC×GC-TOF MS分析。

### 1.4 仪器分析

气相色谱参数:一维色谱柱为Rxi-5MS弱极性气相色谱柱(30 m×0.25 mm×0.25 μm,美国Restek公司),二维色谱柱为Rxi-17Sil中等极性气相色谱柱(1.39 m×0.25 mm×0.25 μm,美国Restek公司)。进样口温度:250 ℃;进样量:1 μL;进样方式:不分流;载气:氦气;流速:1.4 mL/min。升温程序:初始温度为60 ℃,保持1 min,然后以20 ℃/min的速率升至220 ℃,再以5 ℃/min的速率升至290 ℃,保持8 min。二维色谱柱的升温程序高于一维色谱柱5 ℃,调制器保持在比二维色谱柱高15 ℃,调制周期为4 s。

质谱参数:离子化模式:电子轰击离子源(EI, 70 eV);离子源温度:250 ℃;传输线温度:280 ℃;溶剂延迟:4 min;采集模式:全扫描模式;扫描范围:*m/z* 40~580;采集速率:100 spectra/s。各化合物的特征离子、保留时间及内标信息见表1。

### 1.5 方法验证、质量保证与质量控制

在整个样品处理过程中,避免使用塑料容器,减少本底污染。玻璃器皿在使用之前用超纯水和二氯甲烷超声清洗3次,然后在电热恒温鼓风干燥箱中于300 ℃烘干8 h。采用1~500 μg/g混合标准溶液和真实灰尘样品对建立的方法进行了线性关系、检出限(LOD)、准确度和精密度等方面的验证,并通过内标法定量来减小基质效应。回收率试验采用无水硫酸钠作为空白样品,并设置一组流程空白同时进行前处理和测定,每组实验设置3个平行样。根据流程空白和加标样品的测定数据计算不同前处理条件下的回收率。

### 1.6 数据处理

ChromaTOF软件(版本V4.2,美国LECO公司)用于数据采集和处理。该软件可实现自动峰识别、背景扣除、自动基线校正、峰反卷积以及峰面积和体积测定。IBM SPSS 22.0和OriginPro 2023b软件用于统计分析和数据可视化。采用Kruskal-Wallis方差分析和Mann-Whitney检验,分析室内各个功能区的PAEs及其替代品的含量差异,*p*<0.05被认为具有统计学意义。

## 2 结果与讨论

### 2.1 GC×GC-TOF-MS方法的优化

#### 2.1.1 升温程序的优化

为了保证44种目标物良好的分离效果,对GC×GC-TOF-MS的升温程序进行了优化。大部分PAEs及其替代品均含有10个以上的碳原子,多在200 ℃以后出峰,因此本实验选择初始柱温60 ℃保持1 min,然后以较大的升温速率(10 ℃/min)升至200 ℃,再以5 ℃/min的速率升至250 ℃,保持8 min。结果如图1a所示,部分PAEs及其替代品未被检出,且存在组分没有完全分离、二维补偿温度偏低的问题。我们通过调整一维色谱柱的升温程序以及调制器的补偿温度,以增加高沸点PAEs及其替代品的解吸。同时调整二维色谱柱补偿温度,以优化二维保留时间。最终确定升温程序如下:初始温度为60 ℃,保持1 min,然后以20 ℃/min的速率升至220 ℃,再以5 ℃/min的速率升至290 ℃,保持8 min。二维色谱柱补偿温度保持在比一维色谱柱高5 ℃,调节器保持在比二维色谱柱高15 ℃。在此条件下,各组分均被检出,分离效果好,有利于样品的定性定量分析(图1b)。

**图 1:**
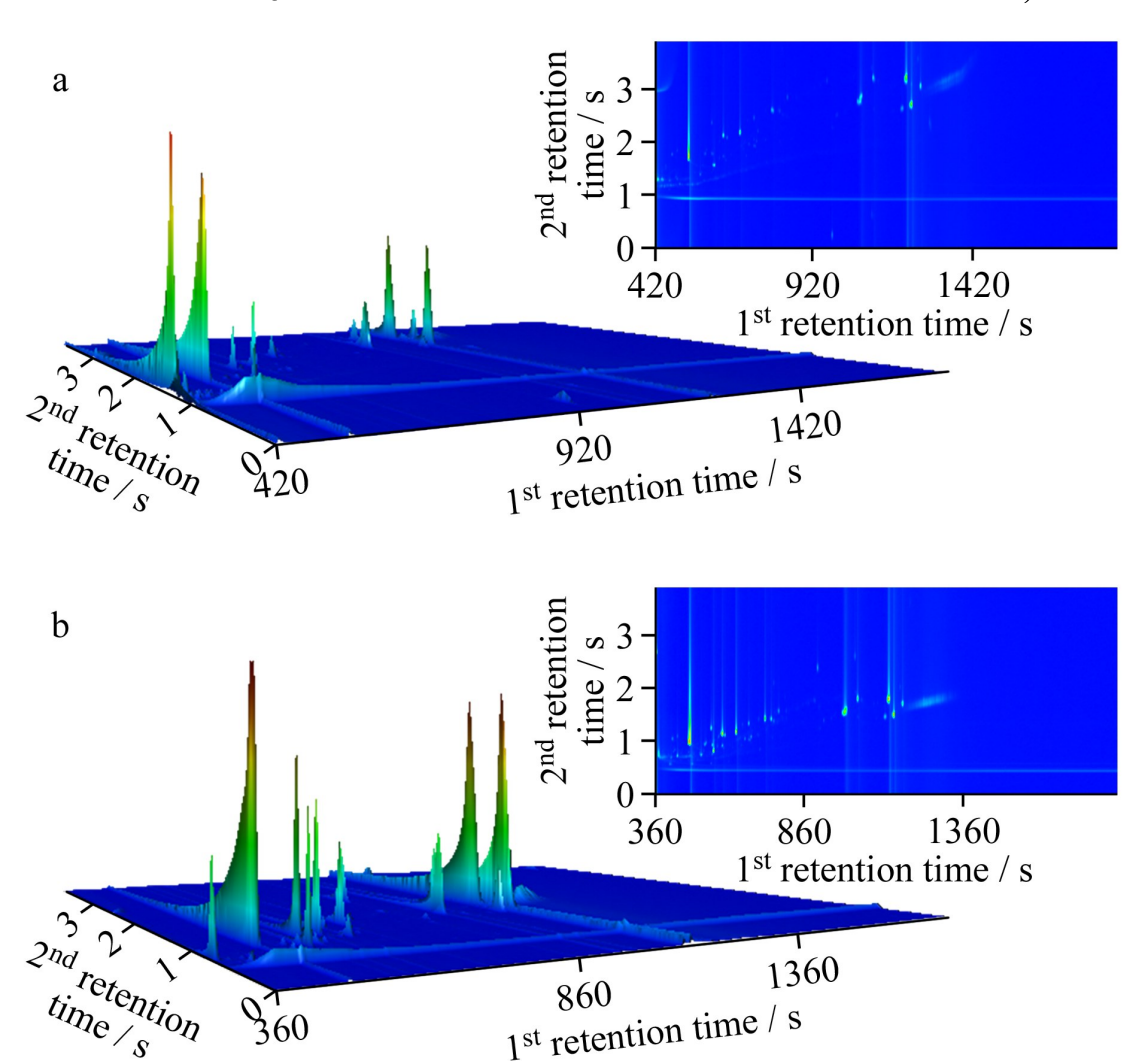
不同升温程序下44种目标物的色谱图

#### 2.1.2 调制周期的优化

调制周期是影响PAEs及其替代品在二维色谱上分离和信号强度的重要参数。调制周期的选择既要保证目标物在一维色谱柱上的分离效果,又要充分发挥二维色谱柱进一步分离的作用。本实验考察了调制周期为3、4和6 s时对目标物的分离度和强度的影响,最终选择调制周期为4 s。如图2所示,调制周期为3 s时,各组分被分割在多个调制周期,导致信号强度降低;调制周期为6 s时,由于降低了一维色谱柱流出峰的调制次数,损失了一维色谱柱的分离度,出现PAEs及其替代品组分共流出的现象;调制周期为4 s时,各组分分离效果好,且二维谱图轮廓清晰。

**图 2:**
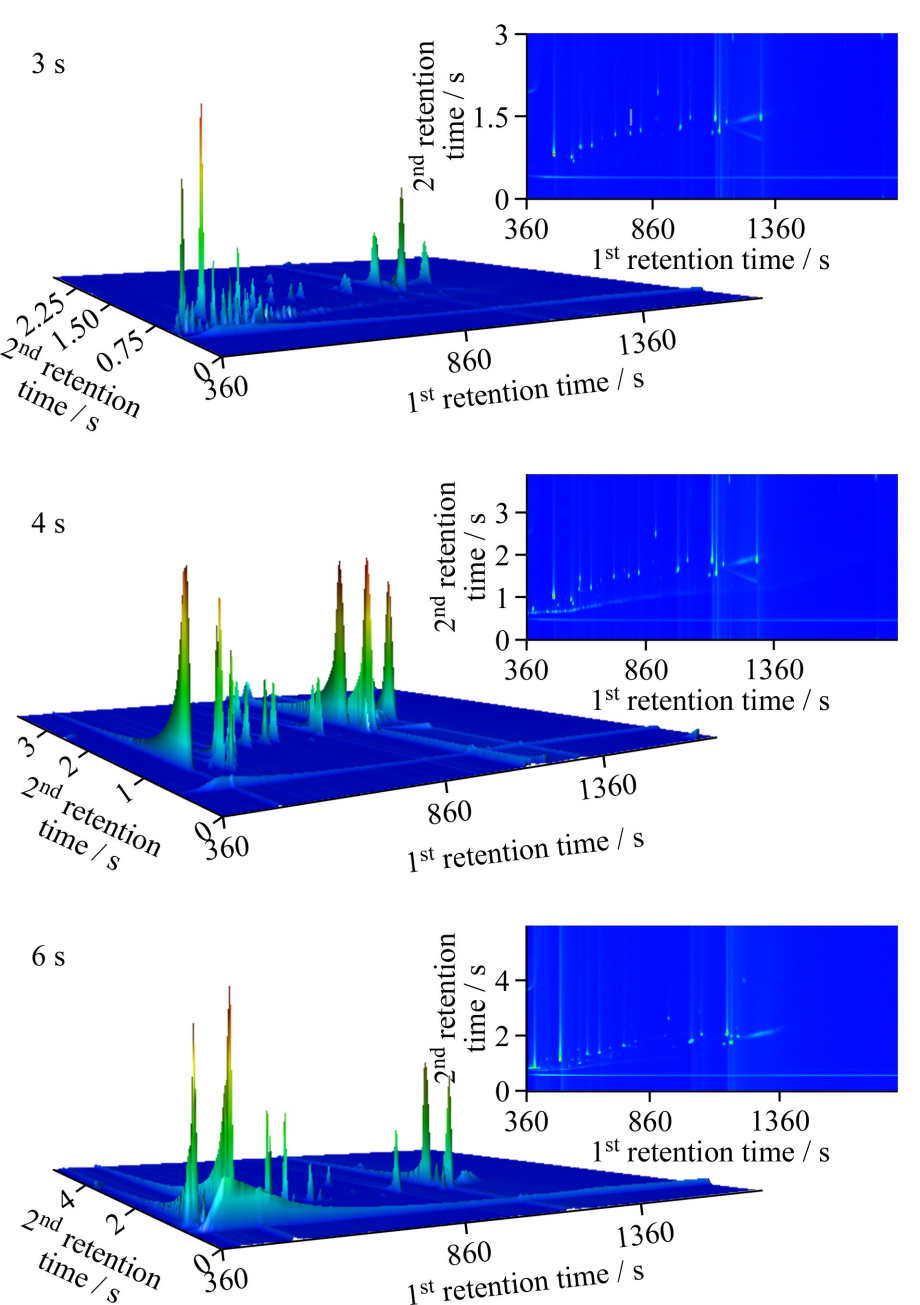
不同调制周期下44种目标物的色谱图

#### 2.1.3 扫描范围的优化

为了保证谱图的完整性,提高分析的灵敏度和准确性,需要选择尽可能窄的质谱扫描范围。本研究选择40作为起始扫描质荷比,使44种目标物的碎片离子均能被扫描到。目标化合物中相对分子质量最大的为TOTM,相对分子质量为547,因此选择580作为扫描的最大质荷比。各化合物的特征离子色谱图见附图S1(https://www.chrom-China.com)。

### 2.2 前处理方法的优化

在超声萃取的过程中,超声萃取溶剂的选取和时间是影响萃取效率的一个重要因素^[36]^。Guo等^[21]^采用丙酮-正己烷混合溶液作为萃取溶剂,但丙酮具有较强的毒性,且不易获取。本研究综合考虑目标物的萃取效果和实验可行性,选择二氯甲烷-正己烷(1∶1, v/v)混合溶液作为萃取溶剂,得到了较好的萃取效果。

为了进一步优化灰尘样品的超声时间,比较了超声时间为10、20、30、40、50和60 min对PAEs及其替代品回收率(加标水平为10 μg/g)的影响。如图3所示,当超声时间为40、50和60 min时,44种目标物的回收率范围分别为70.1%~132%、24%~148%和11.2%~87.5%。结果显示,当超声时间过长时部分PAEs及其替代品的回收率变低。例如,超声时间为60 min时,DiNP、ATBC和DHeNoA的回收率分别为31.8%、36.5%和31.0%。回收率较差的原因可能是长时间的超声处理破坏了PAEs及其替代品的结构,或者在超声的过程中PAEs及其替代品发生了转化。而部分PAEs及其替代品表现出较好的回收率,如DiDP和DiDeA的回收率较好,这可能与其结构相对稳定有关。超声时间为10、20和30 min时,各化合物的回收率范围分别为21.0%~80.1%、71.0%~129%和72.8%~123%。超声时间过短时PAEs及其替代品的回收率变低。例如,超声时间为10 min时,DiPeP和TEC的回收率分别为24.1%和22.0%,这可能是因为较短的超声时间无法将灰尘中的PAEs及其替代品萃取完全。超声时间为30 min时的回收率结果优于20 min,且通过查阅的相关研究发现,超声萃取30 min是大部分研究采用的时间^[34,43,44]^。因此,本研究将超声萃取的时间确定为30 min,各化合物均具有较好的回收率,符合定性定量的分析要求。

**图 3:**
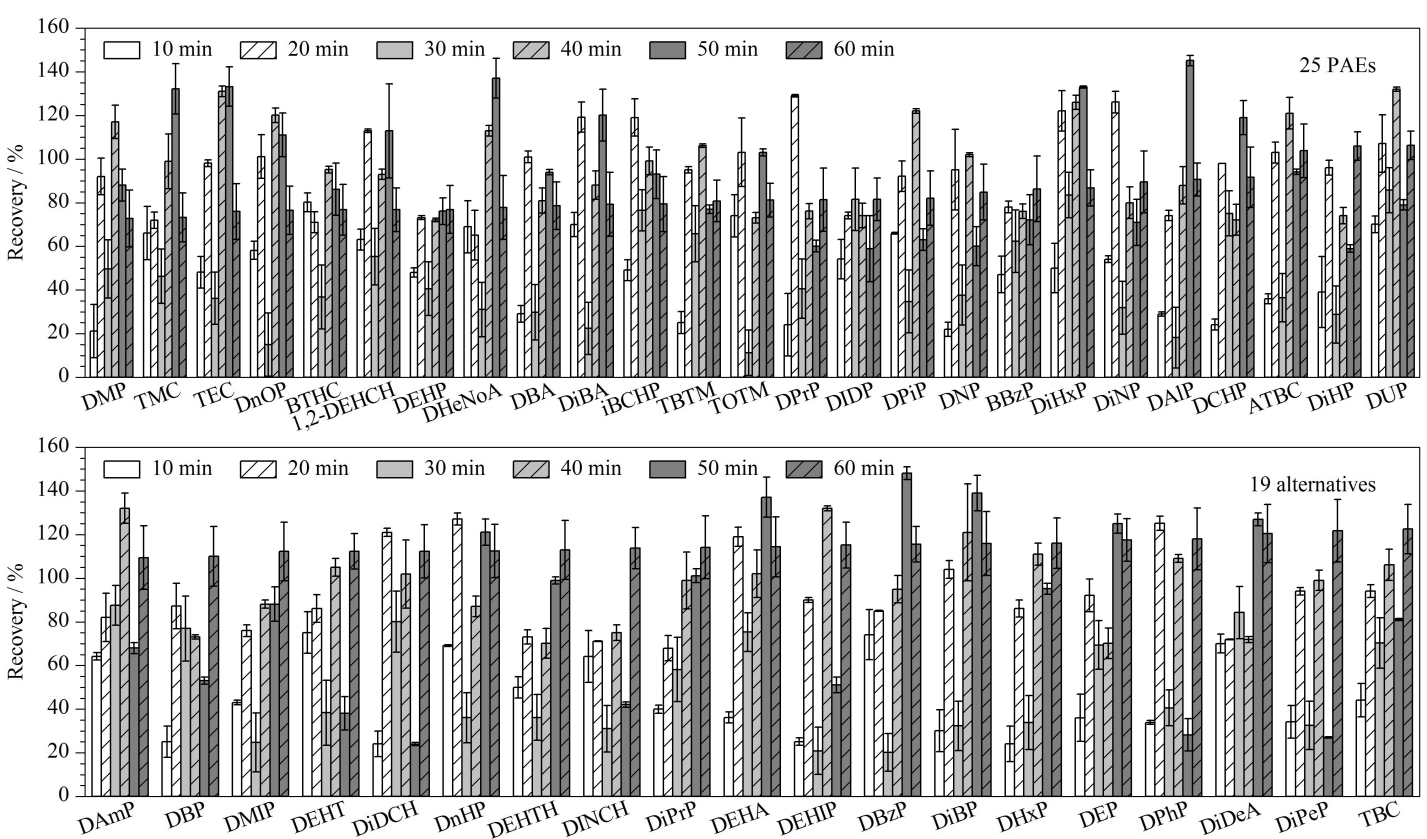
不同超声时间下25种PAEs和19种替代品的回收率(添加水平10 μg/g, *n*=3)

与其他文献中的固相萃取法相比^[4,5,15]^,本研究采用的液液萃取前处理方法具有操作简单、快速、实验成本低的优势。

### 2.3 方法学考察

#### 2.3.1 方法的线性关系和检出限

本研究基于建立的仪器方法,用含量范围为1~500 μg/g的混合标准溶液,以目标物的含量(*x*, μg/g)为横坐标、定量离子的峰面积(*y*)为纵坐标进行定量分析,得到了44种目标物标准曲线的线性拟合方程(附表S1),方程相关系数均大于0.99。无水硫酸钠样品中添加标准溶液,以信噪比(*S/N*)=3和*S/N*=10确定LOD和定量限(LOQ)为0.57~13.0 ng/g和1.71~39.1 ng/g(表2)。

**表2:** 1、10、50 μg/g加标水平下44种目标物的回收率和精密度及其检出限和定量限(*n*=3)

Compound	High (50 μg/g)			Medium (10 μg/g)			Low (1 μg/g)		LOD/ (ng/g)	LOQ/ (ng/g)
	Recovery/%	RSD/%		Recovery/%	RSD/%		Recovery/%	RSD/%		
DEHP	96.1	4.45		76.9	5.84		82.2	13.8	2.31	6.94
BBzP	97.3	11.1		86.3	1.96		108	14.6	2.76	8.29
DBP	99.1	14.9		110	6.19		108	10.5	3.24	9.72
DiBP	96.4	4.82		115	9.17		109	4.97	0.77	2.31
DCHP	97.6	11.4		91.7	9.12		108	6.49	5.69	17.1
DEP	92.2	4.85		117	5.67		125	6.08	1.80	5.42
DHxP	93.5	5.31		116	8.49		87.0	6.66	3.75	11.3
DiHxP	102	8.77		86.8	12.9		107	11.7	3.14	9.43
DMP	95.0	3.50		72.8	1.77		112	4.65	2.16	6.48
DiPrP	108	10.9		114	10.7		100	13.3	1.09	3.25
DPrP	109	8.02		81.4	3.79		77.4	1.74	0.81	2.43
DnHP	102	11.2		113	8.03		101	14.5	2.82	8.46
DiHP	96.1	14.7		106	5.85		107	11.7	7.11	21.3
DnOP	103	6.84		76.5	1.91		111	10.0	9.64	28.9
DNP	99.6	12.0		84.9	3.76		104	13.2	0.96	2.86
DiNP	91.6	7.28		89.5	7.14		88.3	11.1	11.3	33.8
DUP	103	6.58		106	3.30		102	4.14	4.53	13.6
DAlP	90.3	7.43		90.6	5.67		102	6.48	1.07	3.20
DAmP	99.5	4.56		109	8.56		98.7	14.0	0.60	1.79
DiPeP	93.7	2.99		121	4.10		125	6.08	1.06	3.18
iBCHP	99.3	9.82		79.4	1.98		82.0	6.37	2.12	6.35
DPhP	96.8	7.33		118	5.6		88.5	3.96	2.85	8.54
DIDP	95.6	1.68		81.5	12.0		102	7.27	13.0	39.1
DPiP	92.9	2.66		82.1	5.73		120	7.57	9.90	29.7
DBzP	97	6.23		115	6.14		109	12.1	0.57	1.71
ATBC	104	10.5		103	12.1		111	5.88	2.73	8.17
DEHA	104	12.1		114	9.68		109	4.97	4.64	13.9
DHeNoA	105	12.4		77.7	4.74		108	6.47	5.15	15.5
DiBA	102	14.0		79.3	4.83		83.9	10.0	1.47	4.41
DiDeA	107	7.92		120	9.09		112	4.65	0.57	1.71
TOTM	102	10.5		81.1	4.95		106	4.26	2.85	8.54
DEHTH	81.4	1.82		112	9.22		108	10.5	1.71	5.13
DEHIP	99.2	7.15		115	8.84		82.2	13.8	4.29	12.9
TBTM	95.4	4.49		80.8	1.29		84.5	9.74	2.70	8.09
1,2-DEHCH	94.9	5.96		76.8	6.13		117	1.54	2.78	8.33
DEHT	94.3	5.14		113	8.14		77.4	1.74	3.36	10.1
DiDCH	103	8.59		112	9.22		88.3	11.1	2.13	6.40
DMIP	95.1	7.64		112	7.52		103	4.14	4.54	13.6
BTHC	103	7.38		76.8	3.12		102	6.48	0.86	2.57
TMC	88.8	3.66		73.2	1.91		87.2	9.18	8.82	26.5
TBC	104	3.10		123	2.30		77.4	1.74	1.57	4.72
TEC	92.3	3.72		76.0	2.24		101	14.5	1.11	3.34
DBA	90.9	5.24		78.7	5.24		107	11.7	2.76	8.28
DINCH	95.2	5.84		113	6.26		125	6.08	3.33	9.98

#### 2.3.2 方法的准确度和精密度

以不含目标物的无水硫酸钠进行加标回收率试验,添加水平为1、10和50 μg/g,每个加标水平设置3个平行样品,计算平均回收率和相对标准偏差(RSD)。结果如表2所示,25种PAEs的平均回收率范围为72.8%~125%, RSD为1.68%~14.8%(*n*=3), 19种PAEs替代品的平均回收率范围为73.2%~125%, RSD为1.29%~14.5%(*n*=3)。

### 2.4 与其他方法的比较

表3列出了本方法与文献中其他方法在检测灰尘中PAEs及其替代品时PAEs及其替代品的数量和检出限的比较。与其他前处理采用液液萃取方法的文献相比(表3),本方法的整体回收率为72.8%~125%, RSD为1.29%~14.8%,明显优于Tan等^[20]^和Guo等^[21]^的方法,表明该方法的准确度和重复性均较为理想,符合实际样品的分析要求。部分文献采用的传统GC-MS仅对少量的PAEs及其替代品进行了分析^[4,5,15,37,45]^,而本方法采用的GC×GC-TOF-MS可以大幅提高PAEs及其替代品组分的分离,从而对44种目标物同时进行检测和分析,实现了对灰尘中PAEs及其替代品组分的广谱筛查。尽管Tan等^[20]^采用UPLC-MS也可以实现对45种PAEs及其替代品组分的同时检测,但是从方法的灵敏度来看,本方法的检出限范围为0.57~13.0 ng/g,明显优于Tan等的方法(1.50~110 ng/g)。此外,与其他方法相比,本方法的检出限明显低于Deng等^[5]^和Christia等^[15]^的方法,稍逊于Başaran等^[37]^的方法。

**表3:** 本方法与其他分析方法测定室内灰尘中PAEs及其替代品的比较

Analytical method	Number of PAEs and their alternatives	LODs/(ng/g)	Recoveries/%	RSDs/%	Ref.
SPE GC-MS	7	0.16-5.50	77.2-102	9.25-13.8	[4]
LLE GC-MS	12	0.01-0.10	71.3-116	3.44-9.32	[37]
SPE GC-MS	12	2.50-210	71.0-107	<15.0	[5]
LLE GC-MS/MS	9	0.10-0.80	80.0-121	<15.0	[45]
SPE GC-MS	21	100-2000	98.0-130	NA	[15]
LLE GC-MS	9	2.00-10.0	76.0-134	NA	[21]
LLE UPLC-MS	45	1.50-110	75.0-139	NA	[20]
LLE GC×GC-TOF-MS	44	0.57-13.0	72.8-125	1.29-14.8	this method

LLE: liquid-liquid extraction.

### 2.5 方法应用

应用本文建立的方法检测了北京市某高校校园室内灰尘样本(*n*=40)中44种目标物的含量,其中24种物质的检出率大于30%,被进一步分析与讨论。结果如表4和附表S2所示,传统PAEs中DiBP和DEP的检出率为100%,这表明传统PAEs仍然在室内灰尘中分布广泛。替代品中检出率较高的为DBA和TBC,检出率分别为100%和98%。在其他国家的室内灰尘中也发现了TBC等柠檬酸盐类增塑剂的广泛分布^[20]^。这表明PAE替代品已成为增塑剂市场的重要组成部分。室内不同功能区灰尘样本中PAEs及其替代品的总含量范围为2.07~354 μg/g,主要的传统PAEs是DEHP,在实验室、宿舍、食堂和教室中PAEs及其替代品的占比分别为27.4%、24.9%、26.0%和29.5%。DEHP作为一种常用增塑剂,不仅大量应用于电子电器产品的生产中,在其他家用产品中也有广泛的应用^[5]^。这可能是造成DEHP在室内灰尘中大量赋存的原因。DEHTH是室内灰尘中主要的PAEs替代品,在实验室(0.09~108 μg/g)、宿舍(nd~117 μg/g)、食堂(nd~105 μg/g)和教室(nd~100 μg/g)中均具有较高的含量赋存(附表S2)。在之前的研究中也发现了类似的规律^[5,46]^。例如,从瑞典幼儿园收集的粉尘样本中,DEHTH的第95百分位含量高达1500 μg/g^[47]^。

**表4:** 室内灰尘中PAEs及其替代品的检出率、含量均值(*n*=10)和各组分占比

Compound	Laboratory				Dormitory				Canteen				Classroom				Total		
	DF/ %	Mean/ (μg/g)	Proportion/ %		DF/ %	Mean/ (μg/g)	Proportion/ %		DF/ %	Mean/ (μg/g)	Proportion/ %		DF/ %	Mean/ (μg/g)	Proportion/ %		DF/ %	Mean/ (μg/g)	Proportion/ %
DEHP	100	93.8	27.4		80	81.6	24.9		100	75.9	26.0		70	103	29.5		88	354	27.0
DBP	90	25.4	7.42		80	25.7	7.86		100	21.6	7.38		100	21.3	6.11		93	94.0	7.17
DiBP	100	21.2	6.19		100	16.7	5.08		100	17.5	6.00		100	19.5	5.60		100	74.9	5.71
DCHP	90	0.52	0.15		80	0.69	0.21		100	0.45	0.15		60	0.44	0.13		83	2.11	0.16
DEP	100	3.75	1.10		100	2.19	0.67		100	4.13	1.41		100	3.17	0.91		100	13.2	1.01
DHxP	100	1.74	0.51		80	1.95	0.59		100	1.69	0.58		100	1.78	0.51		95	7.16	0.55
DMP	100	22.4	6.57		100	21.2	6.46		100	14.3	4.90		100	16.8	4.81		100	74.7	5.70
DnOP	100	1.76	0.51		100	1.86	0.57		80	2.35	0.80		100	1.51	0.43		95	7.49	0.57
DiNP	70	22.3	6.52		80	25.4	7.74		100	18.8	6.44		60	22.5	6.46		78	89.0	6.79
DUP	100	5.56	1.63		80	5.76	1.76		100	6.47	2.21		70	5.08	1.46		88	22.9	1.74
DPhP	80	6.23	1.82		80	5.12	1.56		70	7.52	2.57		60	6.48	1.86		73	25.4	1.93
DiDP	60	6.38	1.87		100	5.14	1.57		100	7.77	2.66		70	7.94	2.27		83	27.2	2.08
ATBC	100	25.7	7.52		100	33.6	10.3		80	16.8	5.74		100	21.7	6.21		95	97.8	7.46
DEHA	90	1.56	0.46		100	1.59	0.49		100	2.40	0.82		100	1.52	0.43		98	7.06	0.54
DHeNoA	100	3.62	1.06		70	3.87	1.18		100	2.05	0.70		60	4.66	1.34		83	14.2	1.08
DiBA	100	0.59	0.17		80	0.55	0.17		90	0.46	0.16		100	0.47	0.13		93	2.07	0.16
DiDeA	100	5.89	1.72		70	3.99	1.22		100	6.10	2.09		90	4.98	1.43		90	21.0	1.60
TOTM	100	29.0	8.49		100	26.9	8.20		100	30.8	10.5		60	24.2	6.92		90	111	8.46
DEHT	100	0.78	0.23		80	1.69	0.52		100	1.21	0.41		90	1.50	0.43		93	5.18	0.40
DEHTH	100	50.7	14.8		80	50.1	15.3		90	42.9	14.7		60	70.5	20.2		93	214	16.34
BTHC	90	3.02	0.88		60	1.96	0.60		70	2.15	0.74		40	1.94	0.56		65	9.07	0.69
TBC	100	6.82	1.99		100	7.47	2.28		90	5.80	1.99		100	5.84	1.67		98	25.9	1.98
DBA	100	1.07	0.31		100	1.27	0.39		100	1.40	0.48		100	0.72	0.21		100	4.46	0.34
DINCH	100	2.02	0.59		80	1.47	0.45		100	1.72	0.59		60	1.49	0.43		85	6.69	0.51

为了更好地了解PAEs及其替代品在不同室内环境中的污染状况及潜在来源,我们对校园不同环境的PAEs及其替代品含量及组分差异进行了分析(图4a),分析结果表明,教室中DEHTH的含量均显著高于其他室内环境(*p*<0.05)。DEHTH是DEHP的一种主要替代品,教室中DEHTH和DEHP均具有较高的赋存含量,可能来自于环境中的建筑材料和大量桌椅^[48]^。与其他室内环境相似,宿舍环境中的PAEs及其替代品含量也表现出显著差异性。以传统的PAEs为例,DBP在宿舍灰尘中的平均含量为25.7 μg/g,显著高于DBP在其他环境中的赋存含量。先前的研究在个人护理产品中频繁检测到DBP的赋存(检出率为46.3%)^[49]^,造成宿舍中DBP含量较高的原因可能是学生个人护理产品使用量较高^[50]^。食堂是学生用餐的主要场所,这也意味着灰尘中的PAEs及其替代品可能更容易附着在食物上,进而通过手口暴露进入人体,因此分析食堂环境中PAEs及其替代品的赋存状况是十分必要的。除DEHP和DEHTH外,TOTM(30.8 μg/g)是食堂环境中主要的PAEs替代品,且显著高于实验室和教室环境。食堂环境中较高含量的TOTM可能来自于食品包装材料以及手套^[51]^。

**图 4:**
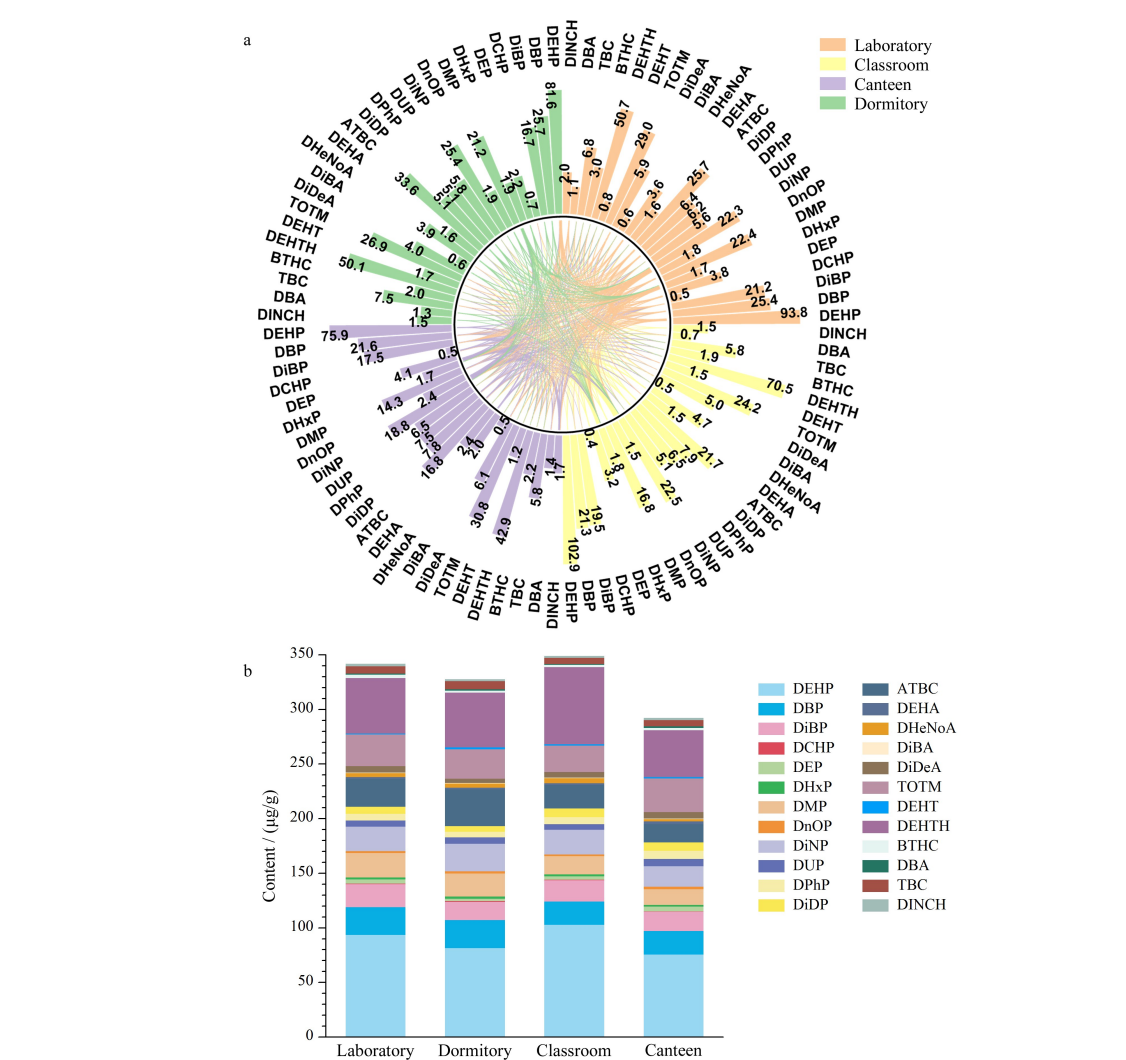
不同室内环境灰尘中主要PAEs及其替代品组分的(a)平均含量比较及(b)含量堆积图(*n*=10)

除了同一种室内环境中PAEs的含量水平存在差异外,同一种PAE或替代品的组成特征在不同区域也具有差异性(图4b)。PAEs替代品中除了占比较高的DEHTH外,ATBC在教室(6.21%)和实验室(7.52%)环境中的占比较大。ATBC作为一种柠檬酸盐类的增塑剂,主要用作功能性或可生物降解聚合物的单体以及洗涤剂或金属处理中的软水剂或络合剂^[20]^。导致教室和实验室中ATBC占比较高的原因可能是频繁地使用洗涤剂清洗地面、桌子、试管、玻璃容器等。相较于其他室内环境,TOTM在食堂(10.5%)灰尘中的占比较高,这可能是来自于食堂连接大量电缆的磨损损耗^[52]^。相比之下,传统PAEs的分布在4个区域之间表现出较小的差异,主要以DEHP为主,其次是DBP、DiBP和DiNP,在其他国家的室内灰尘中也发现了类似的规律^[20]^。

## 3 结论

本研究基于超声萃取和GC×GC-TOF-MS,建立了一种快速、准确、操作简便的方法,可同时分析室内灰尘中44种目标物。本方法具有较高的准确度、精密度和较低的检出限,为室内灰尘中PAEs及其替代品的检测提供了方法,同时为今后识别室内不同功能区中PAEs的来源以及评估室内PAEs的健康风险提供了技术支撑。

## References

[1] Pagoni A, Arvaniti O S, Kalantzi O-I. Environ Res, 2022, 212: 113194 35358548 10.1016/j.envres.2022.113194

[2] Souza J M O, Souza M C O, Rocha B A, et al. Sci Total Environ, 2022, 828: 154486 35278545 10.1016/j.scitotenv.2022.154486

[3] Alp A C, Yerlikaya P. Eur Food Res Technol, 2020, 246(3): 425

[4] Hua L, Guo S, Xu J, et al. Sci Total Environ, 2022, 845: 157251 35817099 10.1016/j.scitotenv.2022.157251

[5] Deng M, Han X, Ge J, et al. J Hazard Mater, 2021, 413: 125322 33588336 10.1016/j.jhazmat.2021.125322

[6] Zheng Y, Li L, Cheng H, et al. Environ Sci Pollut Res, 2022, 29(56): 85547 10.1007/s11356-022-21769-8 35794332

[7] Deng M, Liang X, Du B, et al. Environ Sci Technol Lett, 2021, 8(8): 705

[8] Engel S M, Patisaul H B, Brody C, et al. Am J Public Health, 2021, 111(4): 687 33600256 10.2105/AJPH.2020.306014 PMC7958063

[9] Dong R, Wu Y, Chen J, et al. Chemosphere, 2019, 226: 351 30947045 10.1016/j.chemosphere.2019.03.159

[10] Wang Y, Qian H. Healthcare, 2021, 9(5): 603 34069956 10.3390/healthcare9050603 PMC8157593

[11] Feng W, Liu Y, Ding Y, et al. Arch Toxicol, 2020, 94(4): 1279 32303808 10.1007/s00204-020-02683-9

[12] Ma Y-B, Manzoor R, Jia P-P, et al. Environ Pollut, 2021, 289: 117944 34391046 10.1016/j.envpol.2021.117944

[13] Monti M, Fasano M, Palandri L, et al. Eur J Public Health, 2022, 32: 131226

[14] Eichler C M A, Cohen Hubal E A, Little J C. Environ Sci Technol, 2019, 53(23): 13583 31617344 10.1021/acs.est.9b03794 PMC9311451

[15] Christia C, Poma G, Harrad S, et al. Environ Res, 2019, 171: 204 30665122 10.1016/j.envres.2018.11.034

[16] Bui T T, Giovanoulis G, Cousins A P, et al. Sci Total Environ, 2016, 541: 451 26410720 10.1016/j.scitotenv.2015.09.036

[17] Shi Y, Zhao L, Zhu H, et al. J Hazard Mater, 2023, 459: 132271 37582303 10.1016/j.jhazmat.2023.132271

[18] Efsa Panel on Food Contact Materials E, Aids P, Silano V, et al. EFSA Journal, 2020, 18(1): e05973

[19] Huo C-Y, Li W-L, Liu L-Y, et al. Sci Total Environ, 2023, 863: 160852 36526181 10.1016/j.scitotenv.2022.160852

[20] Tan H, Yang L, Liang X, et al. Environ Sci Technol, 2023, 57(9): 3634 36821817 10.1021/acs.est.2c08110 PMC9996830

[21] Guo Y, Kannan K. Environ Sci Technol, 2011, 45(8): 3788 21434628 10.1021/es2002106

[22] Liu W, Sun Y, Liu N, et al. Indoor Air, 2022, 32(4): e13030 35481931 10.1111/ina.13030

[23] Bu S, Wang Y, Wang H, et al. Build Environ, 2020, 177: 106853

[24] Huang J, Wang X, Guo J, et al. Indoor Air, 2022, 32(11): e13176 36437652 10.1111/ina.13176

[25] Xu H, Musi B, Wang Z, et al. Regul Toxicol Pharmacol, 2019, 104: 50 30826316 10.1016/j.yrtph.2019.02.016

[26] Sheikh I A, Beg M A. Reprod Toxicol, 2019, 83: 46 30468821 10.1016/j.reprotox.2018.11.003

[27] Campioli E, Lau M, Papadopoulos V. Environ Res, 2019, 179: 108773 31605871 10.1016/j.envres.2019.108773

[28] Kambia N K, Séverin I, Farce A, et al. J Appl Toxicol, 2019, 39(7): 1043 30847963 10.1002/jat.3792

[29] Vasconcelos A L, Silva M J, Louro H. J Toxicol Environ Health Sci, Part A, 2019, 82(9): 526 10.1080/15287394.2019.1634376 31242819

[30] Zhou X, Lian J, Cheng Y, et al. Environ Res, 2021, 194: 110681 33428915 10.1016/j.envres.2020.110681

[31] Weng X, Zhu Q, Liao C, et al. Environ Sci Technol, 2023, 57(22): 8189 37196176 10.1021/acs.est.3c00823

[32] Gunathilake T M S U, Ching Y C, Kadokami K. Environ Geochem Health, 2022, 44(3): 677 34170457 10.1007/s10653-021-01013-x

[33] Ye D-M, Yang H, Xu T-T, et al. Environ Sci Technol, 2023, 57(26): 9744 37339114 10.1021/acs.est.3c00932

[34] Bi C, Wang X, Li H, et al. Environ Sci Technol, 2021, 55(1): 341 33287540 10.1021/acs.est.0c05131

[35] Song Z, Shi M, Ren X, et al. J Hazard Mater, 2023, 459: 132202 37562352 10.1016/j.jhazmat.2023.132202

[36] Min K, Weng X, Long P, et al. Talanta, 2021, 231: 122359 33965025 10.1016/j.talanta.2021.122359

[37] Başaran B, Soylu G N, Yilmaz Civan M. Environ Sci Pollut Res, 2020, 27(2): 1808 10.1007/s11356-019-06815-2 31758479

[38] Zhang J, Sun C, Lu R, et al. Environ Res, 2021, 200: 111760 34324846 10.1016/j.envres.2021.111760

[39] Ma J-K, Wei S-L, Tang Q, et al. LWT, 2022, 153: 112426

[40] Dallüge J, Beens J, Brinkman U A T. J Chromatogr A, 2003, 1000(1): 69 12877167 10.1016/s0021-9673(03)00242-5

[41] Stefanuto P-H, Smolinska A, Focant J-F. TrAC-Trends Anal Chem, 2021, 139: 116251

[42] Hurtado-Fernández E, Velázquez-Gómez M, Lacorte S, et al. J Hazard Mater, 2021, 411: 125058 33482505 10.1016/j.jhazmat.2021.125058

[43] Hantzidakis E, Giagkou M, Sakellaris I, et al. Atmosphere, 2023, 14: 418

[44] Al_Qasmi N N, Al-Thaiban H, Helaleh M I H. Environ Sci Pollut Res, 2019, 26: 421 10.1007/s11356-018-3604-8 30406583

[45] Zhu Q, Jia J, Zhang K, et al. Sci Total Environ, 2019, 652: 1187 30586805 10.1016/j.scitotenv.2018.10.326

[46] Zhang Y, Li J, Su G. Environ Sci Technol, 2021, 55(20): 13961 34598436 10.1021/acs.est.1c04402

[47] Larsson K, Lindh C H, Jönsson B A G, et al. Environ Int, 2017, 102: 114 28274486 10.1016/j.envint.2017.02.006

[48] Abdi S, Sobhanardakani S, Lorestani B, et al. Environ Sci Pollut Res, 2021, 28(43): 61151 10.1007/s11356-021-14845-y 34173141

[49] Liu R, Mabury S A. Environ Sci Technol, 2019, 53(22): 13440 31609587 10.1021/acs.est.9b04120

[50] Bao J, Wang M, Ning X, et al. J Toxicol Environ Health Sci, Part A, 2015, 78(5): 325 10.1080/15287394.2014.968696 25734628

[51] De Anda-Flores Y B, Cordón-Cardona B A, González-León A, et al. Food Packag Shelf Life, 2021, 29: 100684

[52] Murawski A, Schmied-Tobies M I H, Rucic E, et al. Environ Res, 2021, 192: 110345 33096061 10.1016/j.envres.2020.110345

